# Acquired Parkinson’s Disease in Alcoholic Cirrhosis: The Rarest Association

**DOI:** 10.7759/cureus.34968

**Published:** 2023-02-14

**Authors:** Apurva Dubey, Sunil Kumar, Sourya Acharya, Vadlamudi Nagendra, Kashish Khurana

**Affiliations:** 1 Department of Medicine, Jawaharlal Nehru Medical College, Datta Meghe Institute of Higher Education & Research, Wardha, IND; 2 Department of Radiology, Jawaharlal Nehru Medical College, Datta Meghe Institute of Higher Education & Research, Wardha, IND; 3 Department of Medicine, Sharda Hospital, Delhi, IND

**Keywords:** acquired hepatocerebral degeneration (ahd), chronic liver disease (cld), manganese, dopamine agonist, parkinsonism

## Abstract

Hepatic encephalopathy is the most common neurologic complication of liver cirrhosis, whereas acquired hepatocerebral degeneration (AHD) is an underappreciated neurologic manifestation. Parkinsonism, ataxia, and neuropsychiatric symptoms are its defining characteristics. In individuals with chronic parenchymal liver disease with portosystemic shunting, it is an underrecognized etiology of psychomotor retardation. It has been hypothesized that the etiology of AHD is due to manganese buildup in the basal ganglia. This case report details a hepatocerebral degeneration (AHD) case in a patient with chronic parenchymal liver disease who improved after taking a dopamine agonist.

## Introduction

Van Woerkom published the first description of acquired hepatocerebral degeneration (AHD) in 1914. Similarly, Victor et al. described the clinical and pathological traits of AHD in their groundbreaking research [1]. Apathy, tremors, psychomotor impairment, and attention impairment are some of the neurological complications. In its basic pathophysiology, it contrasts with Wilson’s disease. According to a theory, portosystemic shunts and liver detoxification failure make the basal ganglia more susceptible to manganese accumulation, which causes AHD; it appears in 0.8-2% of cirrhotic patients [2,3]. Hepatic encephalopathy (HE), a catastrophic complication of cirrhosis and portosystemic shunts, can cause neuropsychiatric symptoms varying from disorientation to coma and can substantially shorten the lifespans of patients. Atypical motor symptoms, including asterixis and irregular tremors, are also common in this condition. Epileptic convulsions are a rare yet severe consequence of HE. AHD is a rare and underdiagnosed condition. Here, we report the case of a 66-year-old man who had a history of longstanding alcoholism and extrapyramidal symptoms, which gradually progressed to psychomotor impairment.

## Case presentation

An elderly male was experiencing resting tremors and balance impairments. He had an episode of seizure accompanied by unconsciousness, incontinence, and a protracted postictal state. He had been in altered sensorium for two weeks. Three months prior, his chronic parenchymal liver disease had been identified. He had a history of chronic alcoholism, consuming 120 mL of country liquor daily for the past eight years. There was no significant family history of neurological or hepatic disorders. On examination, he was afebrile and icteric, with a rhythmic sinus pulse of 78 beats per minute and a blood pressure of 120/80 mmHg. He had hypertonia, increased deep tendon reflexes in all four limbs, and was stuporous upon systemic examination, with bilateral flexors of the plantar reflexes. Except for mild splenomegaly, the examination of other systems was normal. The glabellar tap reflex was elicited through repeated stimulation to the glabellar region of the forehead, as shown in Video 1.

**Video 1:** Demonstration of glabellar tap reflex.

His laboratory parameters on admission are presented in Table 1. The viral markers for hepatitis B and C were negative.

**Table 1:** Laboratory findings of the patient. MCV = mean corpuscular volume; INR = international normalized ratio; PT = prothrombin time; AST = aspartate aminotransferase; ALT = alanine aminotransferase; BUN = blood urea nitrogen

Laboratory parameters	Laboratory results	Reference range
Hemoglobin (g/dL)	8	13.2–16.6
White blood cells (/mm^3^)	9,500	4,500–11,000
Platelets (/mm^3^)	92,000	150,000–450,000
MCV (fL)	74.5	78.9–98.6
INR	1.63	0.8–1.1
PT	11.9	9.4–12.5
AST (U/L)	55	13–39
ALT (U/L)	50	7–52
Alkaline phosphatase (U/L)	99	34–104
Direct bilirubin (mg/dL)	3.0	0–0.2
Indirect bilirubin (mg/dL)	1.5	0.2–0.8
Total bilirubin (mg/dL)	4.5	0.3–1.0
Serum ammonia (μmol/L)	102	9–30
Urea (mg/dL)	29	9–20
Creatinine (mg/dL)	1.4	0.66–1.25
Sodium (mmol/L)	133	137–145
Potassium (mmol/L)	4.4	3.5–5.1
BUN (pg/mL)	16	0–100

He scored 14 on the Model for End-Stage Liver Disease and a Child-Pugh class B (score of 7). Ultrasound of the abdomen and pelvis revealed coarse echotexture of the liver, indicating chronic liver parenchymal disease with portal hypertension. The portal vein diameter was 13 mm, along with mild splenomegaly and mildly raised echotexture of bilateral kidneys, suggestive of grade III renal parenchymal disease. Upper gastrointestinal endoscopy suggested eradicated varix, mild portal hypertensive gastropathy, and mild gastric antral vascular ectasia. First, levetiracetam 500 mg twice daily was used to control the seizures. He began receiving supportive care in addition to anti-hepatic coma treatments such as thiamine supplements, Ryle’s tube feeding, syrup lactulose, and tablet rifaximin. Although the patient’s sensorium did not fully restore, it did improve.

His cerebrospinal fluid examination was within normal range and clear in appearance, opening pressure was 9 cmH_2_O, white cell count was 3 cells/µL, protein was 0.20 g/L, and glucose was 2.6 mmol/L. On day eight, once he became cognizant and ambulatory, his additional clinical evaluation revealed a mask-like visage, monotone voice, and short shuffling stride. Walking with less arm swing was correlated with it.

**Figure 1:**
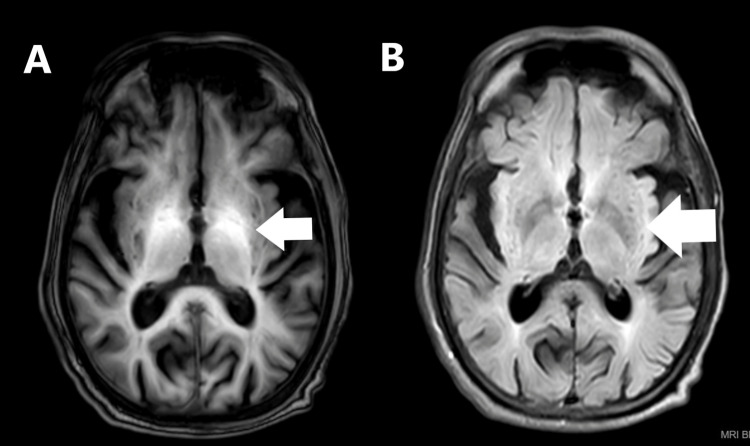
MRI brain axial T1 image (A) at the level of the gangliocapsular region shows symmetrical hyperintensity in bilateral globus pallidus (arrow) and corresponding hypointensity of the bilateral globus pallidus in T2/fluid-attenuated inversion recovery image (B).

**Figure 2:**
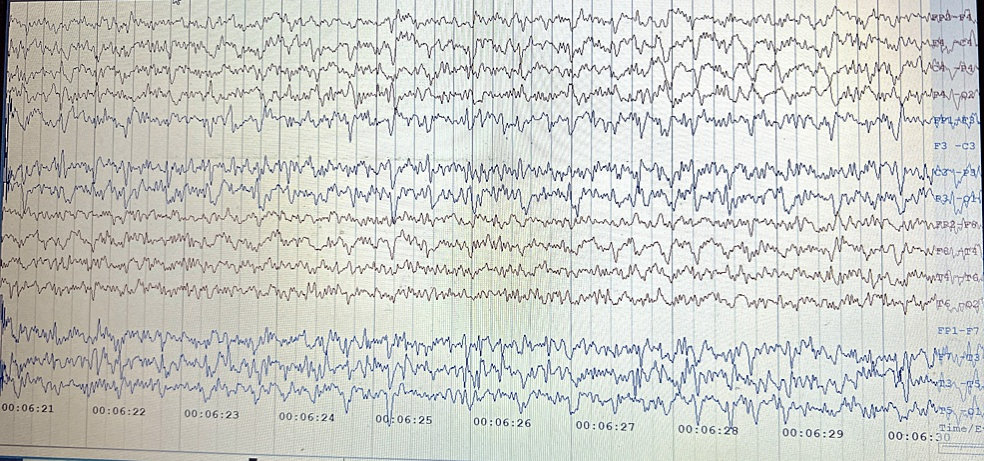
Triphasic waves visible on the electroencephalogram against a slow wave background, indicative of grade II hepatic encephalopathy.

MRI brain axial T1-weighted images at the gangliocapsular region revealed symmetrical hyperintensity in bilateral globus pallidus and corresponding hypointensity in bilateral globus pallidus in T2/fluid-attenuated inversion recovery-weighted image (Figure 1). His electroencephalogram (EEG), recorded with international (10-20) electrode placement, background showed triphasic waveforms with a blunt and broad contour wave activity in the bilateral hemisphere at 7.5 µV (Figure 2). He was placed on the dopamine agonist tablet trihexyphenidyl hydrochloride (2 mg 1/2-0-1/2) and tablet Syndopa (100 + 25 mg 1/2-1/2-1/2) due to the likelihood of AHD. Later, throughout hospitalization, he improved. He was admonished against drinking and advised of liver transplantation. On follow-up, the patient had recovered and could care for himself.

Thus, we encountered a patient with a clinical profile and studies suggesting HE (which partially recovered) with AHD, causing Parkinsonism in the context of chronic liver illness with portal hypertension (ethanol related).

## Discussion

The most prevalent neurological consequence of the severe liver disease is HE. As the illness progresses, the liver’s detoxifying capacity declines. Shunts in the portosystemic system deliver these dangerous compounds to the brain’s bloodstream [4]. Toxic chemicals are deposited in the basal ganglia, which is the pathophysiology of AHD. AHD is a sporadic neurological disease linked to cirrhosis [5]. Neuropsychiatric symptoms and extrapyramidal signs are hallmarks of AHD. Neuropsychiatric signs include apathy, psychomotor retardation, memory loss, and attention difficulties.

Burkhard et al. studied 11 AHD patients over a year, with parkinsonism and cirrhosis being the most common symptoms [6].

The clinical symptoms of AHD have been linked to manganese accumulation in the basal ganglia. The liver must remove manganese from body fluids when performing at its peak excretion level. Manganese is transported to and deposited at the basal ganglia in advanced liver disease with patent portosystemic shunts. As a result, selective neuronal dysfunction occurs, most notably in the basal ganglia, brainstem, cerebral cortex, and surrounding white matter [7]. Extrapyramidal symptoms are brought on by their impact on postsynaptic dopamine receptors and presynaptic dopamine transporters. Manganese deposits exhibit T1 shortening because they are paramagnetic materials. On T1-weighted images, a patient with cirrhosis may display pallidal hyperintensities without neurological symptoms. On T1-weighted images, hyperintensities at the substantia nigra or subthalamic nucleus are a distinctive marker of parkinsonism symptoms. T1-weighted hyperintensities were detected in the subthalamic nucleus and globus pallidus in our patient. In patients with AHD, dopamine agonists produce a mixed response. A significant improvement in the symptoms has been documented following treatment modalities such as portosystemic shunt embolization and liver transplantation. When a dopamine agonist was administered, our patient significantly improved [8-10].

AHD should be suspected when the chronic parenchymal liver disease presents with neurological problems, does not improve with anti-hepatic coma therapy, and has typical signs. According to studies reported in the literature, it responds to liver transplantation, portosystemic shunt embolization, and dopamine agonists.

## Conclusions

Cirrhosis-related parkinsonism might represent a separate, chronic, and widespread subtype of AHD with long-term features distinct from acute HE episodes. Acquired chronic hepatocerebral degeneration is seldom described, and it is frequently misdiagnosed as other neurological diseases, such as HE. In this case report, Parkinson’s disease was the presenting symptom of liver cirrhosis with normal serum ammonia levels. As a result, we have stressed the importance of investigating this condition in patients who develop neurological symptoms after chronic liver disease.
